# Comparison of outcomes between Zero-p implant and anterior cervical plate interbody fusion systems for anterior cervical decompression and fusion: a systematic review and meta-analysis of randomized controlled trials

**DOI:** 10.1186/s13018-022-02940-w

**Published:** 2022-01-25

**Authors:** Tingxin Zhang, Nana Guo, Gang Gao, Hao Liu, Yanhong Li, Feng Gao, Qingxin Zhang, Xiaoyang Tao, Wupeng Yang, Yongjiang Wang

**Affiliations:** 1 Department of Orthopedics, Ordos Central Hospital, 23 Ekin Hollow West Street, Ordos, 017000 China; 2 Critical Care Medicine, Ordos Central Hospital, Ordos, China

**Keywords:** Zero-profile, Anterior cervical plate, Anterior cervical decompression and fusion, Meta-analysis, Systematic review

## Abstract

**Purpose:**

The clinical outcomes of using a zero-profile for anterior cervical decompression and fusion were evaluated by comparison with anterior cervical plates.

**Methods:**

All of the comparative studies published in the PubMed, Cochrane Library, Medline, Web of Science, EBSOChost, and EMBASE databases as of 1 October 2021 were included. All outcomes were analysed using Review Manager 5.4.

**Results:**

*Seven* randomized controlled studies were included with a total of 528 patients, and all studies were randomized controlled studies. The meta-analysis outcomes indicated that the use of zero-profile fixation for anterior cervical decompression and fusion was better than anterior cervical plate fixation regarding the incidence of postoperative dysphagia (*P* < 0.05), adjacent-level ossification (*P* < 0.05), and operational time (*P* < 0.05). However, there were no statistically significant differences in intraoperative blood loss, Visual Analogue Scale, Neck Disability Index, or Japanese Orthopaedic Association scale (all *P* > 0.05) between the zero-profile and anterior cervical plate groups.

**Conclusions:**

The systematic review and meta-analysis indicated that zero-profile and anterior cervical plates could result in good postoperative outcomes in anterior cervical decompression and fusion. No significant differences were found in intraoperative blood loss, Visual Analogue Scale, Neck Disability Index, or Japanese Orthopaedic Association scale**.** However, the zero-profile is superior to the anterior cervical plate in the following measures: incidence of postoperative dysphagia, adjacent-level ossification, and operational time.

*PROSPERO registration* CRD42021278214.

## Introduction

Cervical degenerative disc disease (CDDD) is a common spine disease, and patients with severe symptoms usually require surgical intervention [1]. The anterior cervical decompression and fusion (ACDF) was first described by Smith and Robinson [2] in 1958. The procedure has become the gold-standard operation for CDDD treatment. Traditional ACDF procedures can restore the height of the intervertebral disc and avoid the migration of the implant by applying the anterior cervical plate (ACP) [3]. However, ACDF-related complications such as postoperative dysphagia, adjacent-level degeneration, and soft tissue injury are not rare [4, 5]. In addition, sometimes, it seems to be related to the ACP. Thus, a new stand-alone cervical anterior interbody fusion device, Zero-profile (Zero-p), was designed and developed [6]. Zero-p has a lower profile than an ACP. This type of device can reduce the compression of prevertebral soft tissue and has similar stability and clinical efficacy as ACP. A few meta-analyses have demonstrated that Zero-p in ACDF can produce better or similar outcomes than ACP in ACDF [7–9]. However, all previously published meta-analysis studies had significant limitations, including the absence of randomized controlled studies (RCTs). There is still insufficient level-one evidence to prove the proposed advantages of Zero-p in ACDF. Therefore, we reviewed previous RCTs and conducted this meta-analysis to compare outcomes between Zero-p implants and ACP interbody fusion systems for ACDF.

## Methods

### Literature search strategy

We performed systematic literature searches in six electronic databases, including PubMed, Cochrane Library, Medline, Web of Science, EBSOChost, and EMBASE. We searched using the following combination of MeSH (Medical Subject Heading) terms and free text words: “Zero-profile”, “Zero-p”, “cage and plate”, “anterior cervical disectomy and fusion” and “ACDF”. The search date was from when databases were built to 1 October 2021. We did not restrict searches based on language or publication year. To prevent certain studies from being missed, we manually searched the bibliographies of RCTs, meta-analyses, and systematic reviews.

### Selection of studies

The study inclusion and exclusion processes were divided into two groups. The selection was first based on the title and abstract, and if a decision could not be made from the summary, the full text was retrieved. When there was a disagreement between the two groups, the selection committee was discussed until a consensus was reached.

### Inclusion and exclusion criteria

We included studies that met the following criteria: (1). Included studies were RCTs. (2). A comparative study on the efficacy of Zero-p and ACP in ACDF. (3). The comparison outcomes included at least one of the following: surgical time, intraoperative blood loss, Visual Analogue Scale (VAS), Neck Disability Index (NDI), Japanese Orthopaedic Association (JOA) scale, postoperative dysphagia, and adjacent-level ossification. Studies were excluded according to the following criteria: (1). Editorials, letters, reviews, case reports, and cadaver or animal experiments. (2). The patient was diagnosed with scoliosis, infection or tumour (3). Studies that did not meet the inclusion criteria. (4). The data of the comparison outcomes could not be extracted.

### Data extraction

Two reviewers used standardized data extraction tables. The extracted data included authors, publication date, title, country, study design, follow-up duration, number of patients, mean age of patients, type of operation, and comparison outcomes. The comparison outcomes included surgical time, intraoperative blood loss, VAS, NDI, JOA scale, postoperative dysphagia, and adjacent-level ossification. All data were extracted from article texts, tables, and figures. The research author was contacted for missing data or further information. Two reviewers independently extracted the data; differences were resolved through discussion, and a consensus was reached by including third parties. The data extraction outcomes are shown in Table 1.

**Table 1:** Characteristics of included studies

Author (years)	Country	Study type	Number of samples	Gender (male)	Average age	Follow-up (months)	Surgical level	Outcomes
			ZP/ACP	ZP/ACP	ZP/ACP	ZP/ACP		
Chen (2016) [11]	China	RCT	38/34	25/21	56.2/56.9	36/36	3	4, 5, 6, 7
Li (2015) [12]	China	RCT	23/24	14/12	48.2/49.2	24/24	1	1, 2, 6, 7
He (2018) [13]	China	RCT	52/52	28/27	55.4/59.5	24/24	2 to 4	2, 6
Scholz (2020) [14]	Germany	RCT	21/20	13/11	58/58	24/24	2	3, 4, 5, 6
Xiao (2017) [15]	China	RCT	60/60	33/35	42/43	24/24	1	1, 2, 6
Nemoto (2015) [16]	Japan	RCT	24/22	21/21	40.9/41.6	24/24	1	1, 2, 3, 7
Yan (2016) [17]	China	RCT	49/49	29/29	43.1/43.3	6/6	1 to 2	2, 3, 4, 5, 6

*Outcomes*: 1. Blood loss, 2. Operating time, 3. Visual analogue score, 4. Japanese Orthopaedic Association, 5. Neck Disabled Index, 6. postoperative dysphagia rate, 7. Adjacent-level ossification development rate

*ZP* zero-profile, *ACP* anterior cervical plate, *RCT* randomized controlled trial

### Data analysis

We used Review Manager Version 5.4 (Copenhagen: The Nordic Cochrane Centre, The Cochrane Collaboration) to analyse the data of all outcomes and compare the Zero-p group with the ACP group. For continuous outcomes, such as operating time, intraoperative blood loss, VAS, NDI, and JOA, the means and standard deviations were pooled to a weighted mean difference (WMD) and 95% confidence interval (CI). Risk ratios (RRs) and 95% CIs were used to evaluate dichotomous outcomes, such as postoperative dysphagia and adjacent segment ossification. We used *I*^2^ to quantify heterogeneity. If *I*^2^ > 50%, the heterogeneity was significant, and the unstandardized mean difference was estimated using a random effects model. Otherwise, a fixed effects model was applied.

### Quality assessment

For RCTs, the Cochrane Handbook for Systematic Reviews of Interventions was used [10], including seven domains: random sequence generation, allocation concealment, blinding of participants and personnel, blinding of outcome assessment, incomplete outcome data, selective outcome reporting, and other sources of bias (Fig. 2). Two reviewers independently carried out the quality assessment and discussed disagreements with a third party.

## Results

### Literature search

**Fig. 1:**
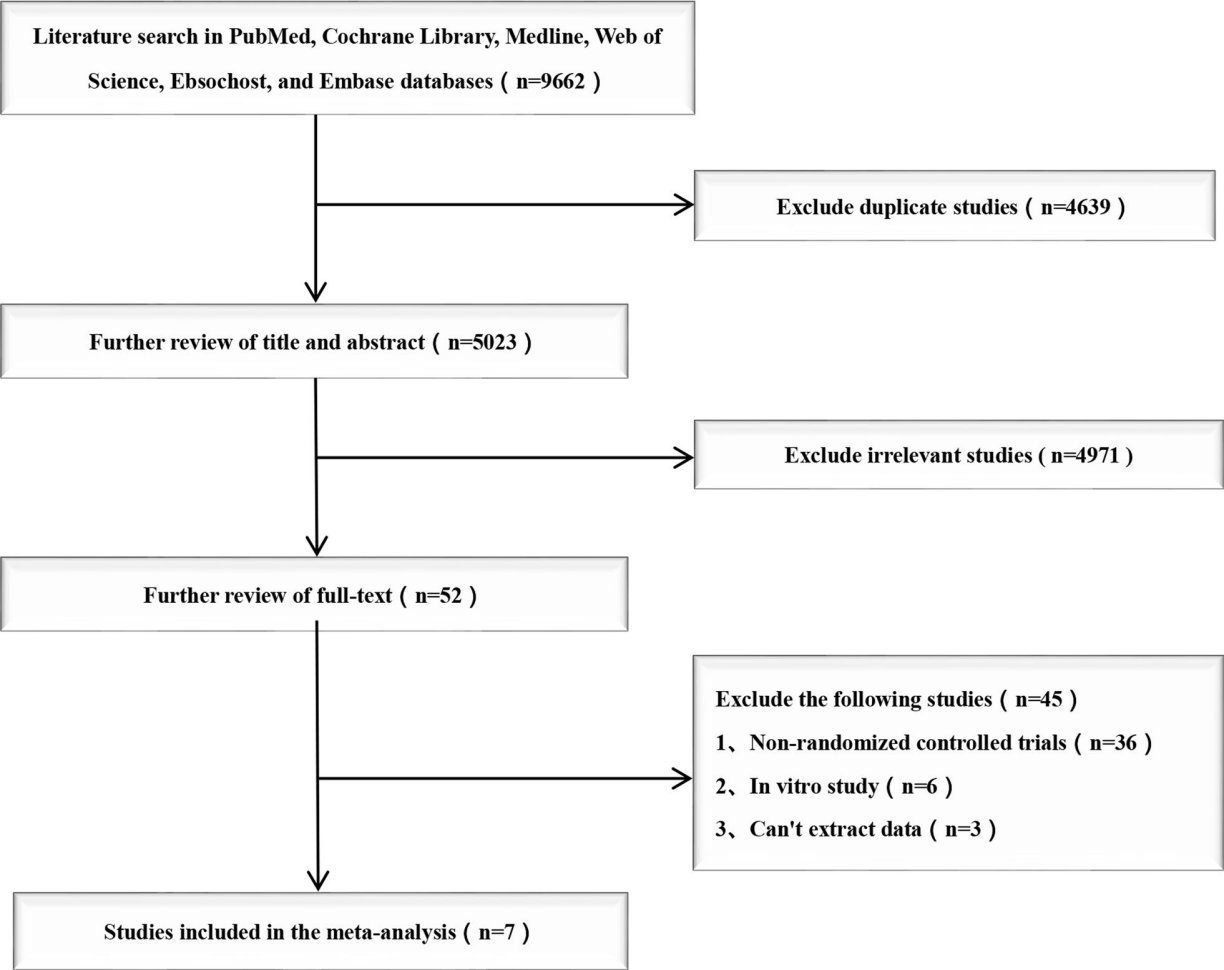
Flow diagram of study selection

**Fig. 2:**
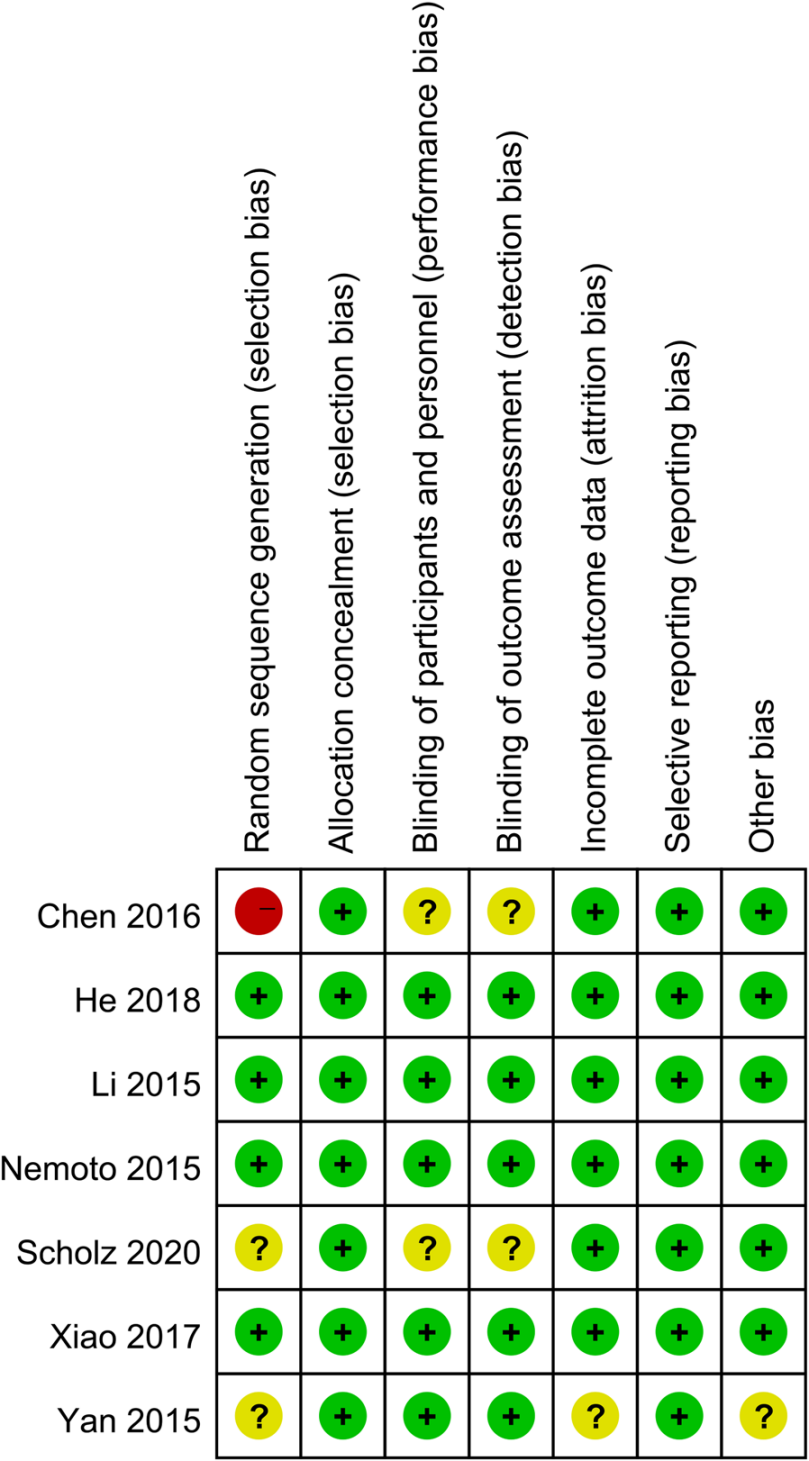
The methodological quality of the randomized controlled trials

There were 9662 studies identified from five electronic databases (Fig. 1). Of those, 4639 studies were duplicates, and 4971 studies were excluded after title and abstract screening. After careful full-text evaluation, seven studies [11–17] were reviewed, and the data were extracted. The demographic and clinical characteristics of the seven studies are described in Table 1. A total of 267 patients who underwent Zero-p were compared with 261 patients who underwent ACP. The mean follow-up time was more than 6 months, and the mean age of the patients was 40–56 years old. Operating times were reported for four studies [13, 15–17]. Intraoperative blood loss was reported for three studies [12, 15, 16]. VAS was reported in three studies [14, 16, 17]. NDI and JOA scores were reported in three studies [11, 14, 17]. Postoperative dysphagia and adjacent segment ossification were reported in six studies [11–15, 17] and three studies [11, 12, 16], respectively (Fig. 2).

### Intraoperative blood loss

**Fig. 3:**
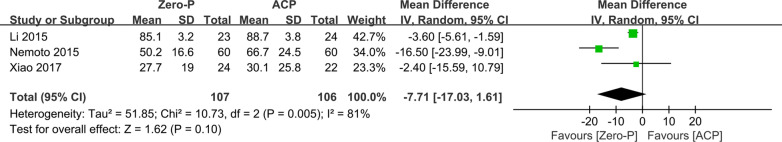
Meta-analysis of Zero-p group versus ACP group in intraoperative blood loss

Three studies [12, 15, 16] with 107 and 106 patients compared the mean intraoperative blood loss between the Zero-p and ACP groups. The meta-analysis indicated no significant differences between the Zero-p and ACP groups (WMD, − 7.71; 95% CI, − 17.03 to 1.61; *P* > 0.05). The heterogeneity test outcome (*I*^2^ = 81%) indicated significant heterogeneity (Fig. 3).

### Operating time

**Fig. 4:**
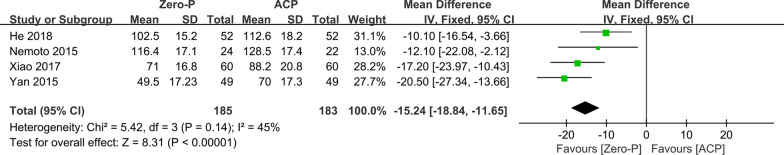
Meta-analysis of Zero-p group versus ACP group in operating time

Four studies [13, 15–17] with 185 and 183 patients compared the mean operating time between the Zero-p and ACP groups. We divided the operation time > 100 min and < 100 min into two subgroups for meta-analysis. In the > 100 min subgroup, the Zero-p group had significantly fewer operating times than the ACP group (WMD, − 10.69; 95% CI, − 16.10 to − 5.27; *P* < 0.05). In the < 100 min subgroup, the Zero-p group had significantly fewer operating times than the ACP group (WMD, − 18.83; 95% CI, − 23.64 to − 14.02; *P* < 0.05). The pooled outcomes showed that the Zero-p group had significantly fewer operating times than the ACP group (WMD, − 15.24; 95% CI, − 18.84 to − 11.65; *P* < 0.05). The heterogeneity test outcome (*I*^2^ = 45%) and the fixed effects model were applied. The results showed that the use of Zero-p in ACDF can significantly reduce the operating time compared with ACP (Fig. 4).

### VAS

**Fig. 5:**
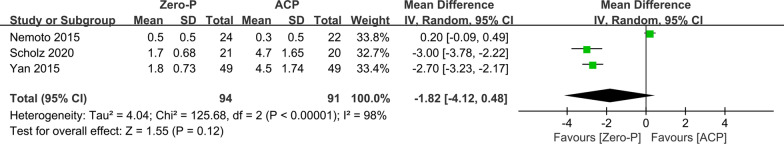
Meta-analysis of Zero-p group versus ACP group inVAS

Three studies [14, 16, 17] with 94 and 91 patients, respectively, compared the mean VAS between the Zero-p and ACP groups. The meta-analysis indicated no significant differences between the Zero-p and ACP groups (WMD, − 1.82; 95% CI, − 4.12 to 0.48; P > 0.05). The heterogeneity test outcome (*I*^2^ = 98%) demonstrated significant heterogeneity. (Fig. 5).

### NDI and JOA scores

**Fig. 6:**
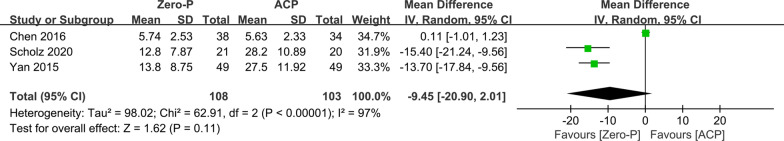
Meta-analysis of Zero-p group versus ACP group in NDI

Three studies [11, 14, 17] with 108 and 103 patients, respectively, compared mean NDI scores between the Zero-p and ACP groups. The meta-analysis concluded no significant differences between the Zero-p and ACP groups (WMD, − 9.45; 95% CI, − 20.9 to − 2.01; *P* > 0.05). The heterogeneity test outcome (*I*^2^ = 79%) has significant heterogeneity (Fig. 6).

**Fig. 7:**
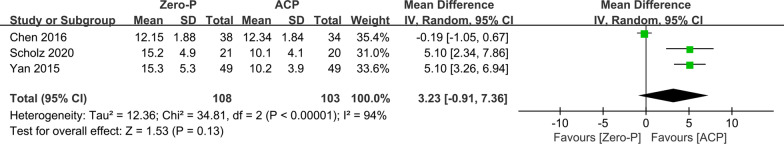
Meta-analysis of Zero-p group versus ACP group in JOA

Three studies [11, 14, 17] with 108 and 103 patients, respectively, compared mean JOA scores between the Zero-p and ACP groups. The meta-analysis indicated no significant differences between the Zero-p and ACP groups (WMD, 3.23; 95% CI, − 0.91 to 7.36; *P* > 0.05). The heterogeneity test outcome (*I*^2^ = 94%) indicated significant heterogeneity (Fig. 7).

### Postoperative dysphagia

**Fig. 8:**
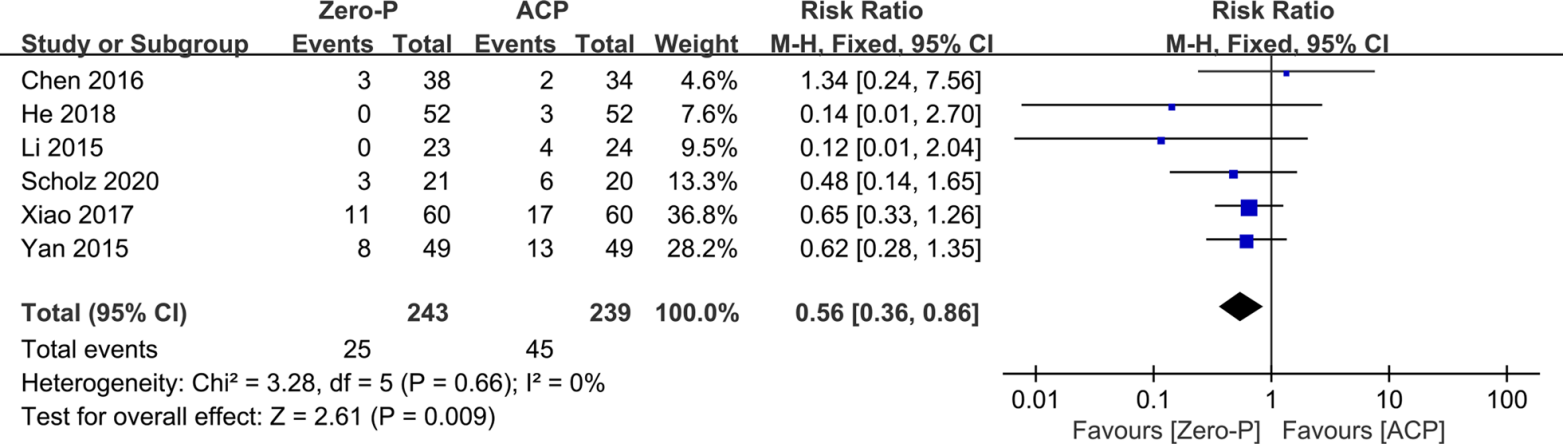
Meta-analysis of Zero-p group versus ACP group in postoperative dysphagia

Six studies [11–15, 17] with 243 and 239 patients, respectively, compared the incidence of postoperative dysphagia between the Zero-p and ACP groups. The pooled outcomes indicated that the Zero-p group had a significantly lower incidence of postoperative dysphagia than the ACP group (RR, 0.56; 95% CI, 0.36 to 0.86; *P* < 0.05). The heterogeneity test outcome was *I*^2^ = 0, and the fixed effects model was applied (Fig. 8).

### Adjacent-level ossification

**Fig. 9:**
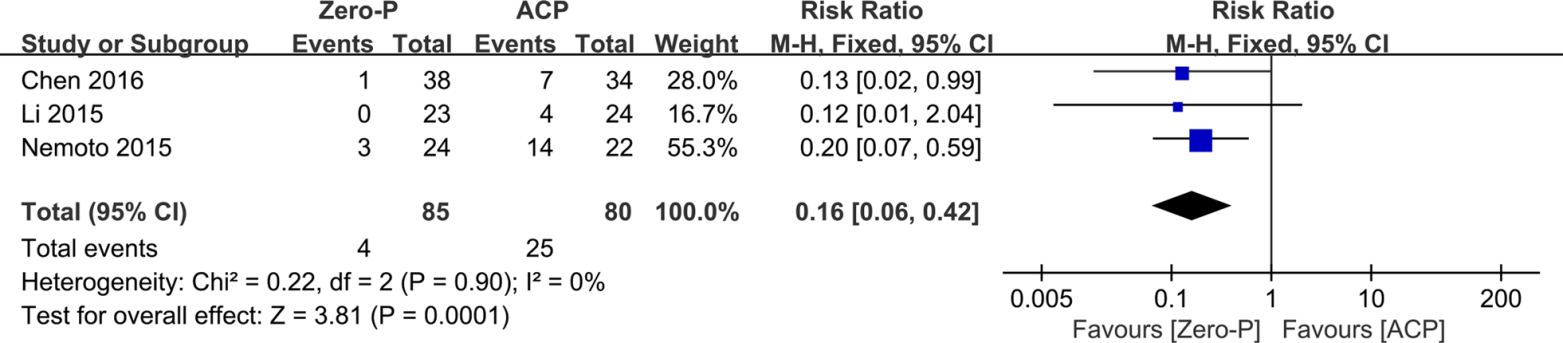
Meta-analysis of Zero-p group versus ACP group in adjacent-level ossification

Three studies [11, 12, 16] with 85 and 80 patients, respectively, compared the incidence of adjacent-level ossification between the Zero-p and ACP groups. The pooled outcomes noted that the Zero-p group had a significantly lower incidence of adjacent-level ossification than the ACP group (RR, 0.16; 95% CI, 0.06 to 0.42; *P* < 0.05). The heterogeneity test outcome was *I*^2^ = 0, and the fixed effects model was applied (Fig. 9).

## Discussion

ACDF is a widely accepted surgical method for degenerative cervical spine diseases treatment [18]. This procedure can decompress the spinal cord, affect nerve roots and improve the stability of the cervical spine. During ACDF, an ACP is usually applied to enhance cervical stability, increase the interbody fusion rate and prevent graft dislocation or subsidence [19]. However, some postoperative complications seem to be related to ACP, such as oesophageal soft tissue damage, neurovascular injuries, and dysphagia [20]. The zero-profile implant is an independent anchoring spacer designed to minimize these complications, avoiding contact between the implant and the anterior soft tissue while providing the spine a stable biomechanical environment [21]. Several relevant studies comparing Zero-p and ACP in ACDF for degenerative cervical spondylosis patients have been reported. However, there is still a debate about whether Zero-p is better than ACP.

In our meta-analysis, the information of 528 patients was extracted from seven published RCTs using the Cochrane Handbook for Systematic Reviews of Interventions for quality assessment. The outcomes indicated that the included literature was of high quality. Our study demonstrated that the operation time, the incidence of postoperative dysphagia, and adjacent segment ossification of Zero-p in ACDF were significantly lower than those of ACP fixation. For intraoperative blood loss, VAS and NDI scores, and JOA scores, the meta-analysis outcomes have no significant differences between the Zero-p group and the ACP group.

Regarding the operating time, Chang et al. [22] reported that the operation time of Zero-p in ACDF is shorter than that of ACP fixation. Lan et al. indicated that there was no significant difference in terms of operation time between Zero-p and ACP [23]. We showed that the use of Zero-p can significantly reduce the operating time compared with ACP fixation, which is likely due to Zero-p saving time to harvest the autologous iliac graft. Moreover, since there is no steel plate, there is no need to polish the bone spurs on the anterior edge of the vertebral body, which reduces the operation time. Because Zero-p has no steel plate, there is no need to polish the bone spurs on the anterior vertebral body, thus reducing the operation time. Nemoto et al. [16] reported that Zero-p has a one-step locking mechanism with the simple insertion of the cage and tightening the screws, thus shortening the operation time.

Postoperative dysphagia is one of the most common complications after ACDF. Although we currently do not know the mechanism of dysphagia after ACDF, some hypotheses have been proposed. Lee et al. [24] noted a positive correlation between plate thickness and postoperative dysphagia rate. Joaquim et al. [25] showed that the causes of dysphagia after ACDF include postoperative soft tissue oedema, oesophageal injury, postoperative haematoma, and surrounding soft tissue adhesions. The Zero-p device can be inserted into the intervertebral space to avoid direct stimulation of the oesophagus and reduce oesophageal adhesions. Yang et al. noticed that the incidence and severity of dysphagia in the Zero-p group were lower than those in the ACP group [26]. Miao et al. [27] also obtained a similar outcome. Shao et al. meta-analysis [28] concluded that the Zero-p group was associated with a lower incidence of dysphagia at postoperative 1, 3, and 6 months and at the final follow-up than the ACP group. The meta-analysis of Sun et al. [29] also indicated that the incidence and severity of dysphagia in the Zero-p group were lower than those in the ACP group. However, most of the studies selected in their meta-analysis were not RCTs. In our meta-analysis, six randomized controlled studies reported dysphagia after the procedure. The results indicated that the Zero-p group had a significantly lower incidence of postoperative dysphagia than the ACP group (*P* < 0.05).

Another common complication of ACDF is adjacent-level ossification. Park et al. [30] indicated a positive association between adjacent-level ossification following anterior cervical plate procedures and the plate-to-disc distance. Huang et al. [31] also indicated that anterior plates with ACDF were associated with adjacent-level ossification. They reported that plate-to-disc distance < 5 mm was significantly associated with adjacent-level ossification. Yang et al. [26] demonstrated that Zero-p can reduce the incidence of adjacent-level ossification and the plate length, which also affects the incidence of adjacent-level ossification. Lee et al. [32] showed that using a shorter plate with longer angulated screws resulted in a significantly reduced incidence of adjacent-level ossification. In our meta-analysis, three randomized controlled studies reported dysphagia after the procedure. The results indicated that the Zero-p group had a significantly lower adjacent-level ossification incidence than the ACP group (*P* < 0.05). Kim et al. [33] indicated that adjacent-level ossification may be caused by ossification due to the inflammatory reaction between the plate and the anterior longitudinal ligament. Although the aetiology of adjacent-level ossification is not known, many scholars are still concerned and suggest avoiding adjacent-level ossification.

## Conclusion

Based on our analysis, Zero-p implants can reduce the incidence of postoperative dysphagia and adjacent-level ossification and reduce the operation time compared with ACP implants. However, for intraoperative blood loss, VAS, JOA score and NDI score, Zero-p implant, and ACP implant achieved similar clinical outcomes. Based on current evidence, we suggest that the Zero-p implant should be used in ACDF if possible to reduce the incidence of postoperative dysphagia and adjacent-level ossification.

## Data Availability

All data generated or analysed during this study are included in this published article and its supplementary information files.

## Abbreviations

CDDD: Cervical degenerative disc disease
Zero-p: Zero-profile
ACP: Anterior cervical plate
NDI: Neck Disability Index
VAS: Visual Analogue Scale
JOA: Japanese Orthopaedic Association
RCTs: Randomized controlled trials
WMD: Weighted mean difference
CI: Confidence interval
RRs: Risk ratios
ACDF: Anterior cervical decompression and fusion
